# Clinical profiles of patients colonized or infected with extended-spectrum beta-lactamase producing *Enterobacteriaceae *isolates: a 20 month retrospective study at a Belgian University Hospital

**DOI:** 10.1186/1471-2334-11-12

**Published:** 2011-01-12

**Authors:** Didier Schoevaerdts, Pierre Bogaerts, Alexandre Grimmelprez, Marie de Saint-Hubert, Bénédicte Delaere, Jacques Jamart, Christian Swine, Youri Glupczynski

**Affiliations:** 1 Department of Geriatric Medicine, Cliniques Universitaires UCL de Mont-Godinne, 5530 Yvoir, Belgium; 2 Department of Microbiology and Infection Control Unit, Cliniques Universitaires UCL de Mont-Godinne, 5530 Yvoir, Belgium; 3 Scientific Support Unit, Biostatistics, Cliniques Universitaires UCL de Mont-Godinne, 5530 Yvoir, Belgium

## Abstract

**Background:**

Description of the clinical pictures of patients colonized or infected by ESBL-producing *Enterobacteriaceae *isolates and admitted to hospital are rather scarce in Europe. However, a better delineation of the clinical patterns associated with the carriage of ESBL-producing isolates may allow healthcare providers to identify more rapidly at risk patients. This matter is of particular concern because of the growing proportion of ESBL-producing *Enterobacteriaceae *species isolates worldwide.

**Methods:**

We undertook a descriptive analysis of 114 consecutive patients in whom ESBL-producing *Enterobacteriaceae *isolates were collected from clinical specimens over a 20-month period. Clinical data were obtained through retrospective analysis of medical record charts. Microbiological cultures were carried out by standard laboratory methods.

**Results:**

The proportion of ESBL-producing *Enterobacteriaceae *strains after exclusion of duplicate isolates was 4.5% and the incidence rate was 4.3 cases/1000 patients admitted. Healthcare-associated acquisition was important (n = 104) while community-acquisition was less frequently found (n = 10). Among the former group, two-thirds of the patients were aged over 65 years and 24% of these were living in nursing homes. Sixty-eight (65%) of the patients with healthcare-associated ESBL, were considered clinically infected. In this group, the number and severity of co-morbidities was high, particularly including diabetes mellitus and chronic renal insufficiency. Other known risk factors for ESBL colonization or infection such as prior antibiotic exposure, urinary catheter or previous hospitalisation were also often found. The four main diagnostic categories were: urinary tract infections, lower respiratory tract infections, septicaemia and intra-abdominal infections. For hospitalized patients, the median hospital length of stay was 23 days and the average mortality rate during hospitalization was 13% (Confidence Interval 95%: 7-19). *Escherichia coli*, by far, accounted as the most common ESBL-producing *Enterobacteriaceae *species (77/114; [68%]) while CTX-M-1 group was by far the most prevalent ESBL enzyme (n = 56).

**Conclusion:**

In this retrospective study, the clinical profiles of patients carrying healthcare-associated ESBL-producing *Enterobacteriacae *is characterized by a high prevalence rate of several major co-morbidities and potential known risk factors. Both, the length of hospital stay and overall hospital mortality rates were particularly high. A prospective case-control matched study should be designed and performed in order to control for possible inclusion bias.

## Background

Antimicrobial resistance is a major public health concern in human medicine both in the community and in medical institutions [1]. Aged patients, especially frail elderly, constitute a subgroup of persons which seems more affected than other population groups. For instance, the carriage of methicillin-resistant *Staphylococcus aureus *(MRSA) in acute care hospitals has been well documented in Belgium since the early nineties [2]. Over the last years, large reservoirs of MRSA carriers have been identified among elderly subjects living in nursing homes [3]. A recent cross-sectional survey carried-out in 2005 among 60 Belgian nursing homes (NH) showed an average prevalence rate of MRSA carriage as high as 20% of the residents (with ranges between 2 and 42%) [4]. By contrast, epidemiologic surveys of multi-drug resistant gram-negative microorganisms (MDR) have been very scarce and the current prevalence of such organisms currently remains only partially known in most European structures of care, especially in long-term care facilities [5-7]. Initial reports of ESBL-producers in the eighties and early nineties essentially focused on the descriptions of nosocomial outbreaks, mainly in intensive care unit settings, caused by *Klebsiella *and *Enterobacter *spp. which produced various TEM- or SHV- ESBL subtypes [8]. More recently, novel ESBLs coding genes (e.g. of the CTX-M family) derived from plasmid mobilization of environmental or soil organisms have emerged worldwide in several enterobacteria species particularly in *E. coli *[9]. The CTX-M ESBLs are most often encountered in association with community-acquired infections rather than with hospital outbreaks [8]. In a nationwide surveillance program, the proportion of ESBL-producing *E. coli *isolated from clinical specimens referred to hospital-based laboratories was found to rise from 3.6% in 2005 to 4.8% in 2008 [10,11].

Reports depicting the clinical pictures and profiles of patients colonized or infected by ESBL-producing *Enterobacteriaceae *isolates and admitted to hospital have mainly been published from the United States, Canada or Israel but are rather limited in Europe [8,9,12]. In order to gain a better insight of the epidemiology and the risk factors associated with ESBLs, we undertook a descriptive analysis of the clinical charts of patients in whom ESBL-producing microorganisms had been isolated from clinical samples.

## Methods

### Study Population

The study took place at Mont-Godinne hospital, Catholic University of Louvain - UCL, a 370-bed tertiary-care teaching hospital located in a rural area in the southern part of Belgium. This hospital mainly provides acute care diagnostic and treatment services including cardio-vascular surgery, chronic respiratory diseases and onco-haematology and it comprises 27 beds of geriatric medicine as well as 20 beds of rehabilitation. Ambulatory care, including one-day clinic and geriatric day hospital are also provided.

### Study Design

In this retrospective descriptive survey carried-out over a 20-month period between January 2008 and August 2009, 114 patients were found to be colonized or infected with one ESBL-producing *Enterobacteriaceae *isolates. This study took part in the setting of a comprehensive program aimed to improve hygiene and infection control locally. Despite the limitation to its retrospective character, this study was undertaken because it was estimated that it could provide easily interesting clinical and microbiological informations in patients colonized or infected with an ESBL-producing microorganism before planning a prospective study. Since all isolates were considered sporadic and because no nosocomial outbreaks were occurring at the time of the study, no systematic screening for ESBL carriage was performed upon admission to hospital.

### Data Collection

All patients' demographic and microbiological data were recorded in an electronic database. Clinical data were collected by retrospective analyses of computerized medical charts.

**Clinical data **included several demographics variables such as age, gender and geographic area of residency. Significant co-morbidities (diabetes mellitus, chronic renal insufficiency, congestive heart failure, chronic respiratory disease, liver disease, haematological malignancies) were collected using the Cumulative Illness Rating Scale for Geriatrics (CIRS-G) [13]. This scale is used for a comprehensive review of medical problems by organ systems, based on a 0 through 4 rating, yielding a cumulative score. Every single disease has to be classified in one of the 14 appropriate systems and rated according to its severity: 0: past problem without clinical relevance, 1: past significant problem or current mild problem, 2: moderate disability or first line therapy needed, 3: severe problem with constant disability or hard to control chronic problems, 4 extremely severe problem or immediate treatment required or severe functional impairment. For this scale, we choose to report results using the mean global score (which in theory can vary from 0 to 56) and the mean number of different categories of affected systems (from 0 to 14 systems) [14]. For hospitalised patients, the mean Norton index was recorded from the medical charts selecting the score that was measured closest to the date of the first identification of the ESBL [15,16]. The Norton scale is a risk assessment scale used for prediction of pressure sore development among elderly patients. The scale included five variables rated from 0 to 4 (general medical condition, mental state, level of activity, level of mobility and incontinence). With a maximum score of 20, a cut-off below 14 has been used to predict patients at risk of pressure sores.

We classified patients in two different groups according to the presence of microbiological colonization by an ESBL-producing organism only or to the occurrence of presumed or proven infection. Colonization was defined by the absence of clinical signs of active infection and by the absence of antibiotic administration during the hospital stay after the detection of an ESBL-producing isolate. Infections were categorized by anatomical sites as well as by their most probable origin of acquisition (community- versus healthcare-setting). Healthcare-associated acquisition of an ESBL-producing *Enterobacteriaceae *was defined as follow: residency in a nursing home before admission, hospitalization within the previous 12 months or multiple contacts with the hospital within the previous year (≥ 3 contacts within the previous 12 months including patients in ambulatory care clinic or attendance to the emergency room). Community-acquisition referred to organisms that were isolated from clinical specimens of non-hospitalized patients and in the absence of any of the criteria used for healthcare-acquisition.

Putative known risk factors for carriage of multi-drug resistant pathogens were also recorded: prior exposure to antimicrobial drugs (defined as a categorical variable, e.g. exposure or no exposure), hospitalization within the previous 12 months, duration of stay exceeding 8 days (the average duration of hospital stay at our hospital), previous hospitalization in the intensive care unit (ICU), previously known MRSA carriage, pressure sores and/or wounds, and presence of chronic catheters (urinary catheter, percutaneous gastrostomy and tracheotomy) [9].

**Microbiological data **included the anatomic sites of isolation, species identification, the type of ESBL as well as the antimicrobial in vitro susceptibility and resistance patterns.

**Patient's outcomes **were defined (for inpatients only) as the mortality rate during hospitalization and the median length of hospital stay (LOS).

### Microbiological analyses

Bacterial isolates were identified by conventional microbiological methods and tested for in vitro susceptibility using the disk diffusion method according to Clinical Laboratory Standards Institute (CLSI ) guidelines [17] and by automated Vitek 2 system (AST-046 susceptibility cards) (bioMérieux, Marcy l'Étoile, France). The presence of ESBL was detected by a two-step approach as per recommendations of the CLSI [17]: i.e. screening of resistance to 3^rd ^generation cephalosporins by MIC and/or disk diffusion testing and by phenotypic confirmatory tests for the presence of ESBLs (disc diffusion and/or MIC susceptibility testing of 3^rd ^generation cephalosporins in the presence of clavulanic acid). For AmpC-inducible species, a double combination disc test with ceftazidime and cefotaxime alone or in the presence of clavulanic acid was performed in the presence of phenylboronic acid (400 μg) spotted on discs and by cefepime, alone and with clavulanic acid, as an additional test [18]. A difference in diameters of ≥ 5 mm in the presence of clavulanic acid was deemed as a positive ESBL result.

Detection and characterization of ESBLs was carried out by a commercial multiplex ligation PCR microarray (Check-Points^®^, Wageningen, The Nederlands) targeting the most common ESBL coding genes (i.e. *bla*_TEM_, *bla*_SHV _and *bla*_CTX-M_) [19].

### Statistical analysis

Numerical data are expressed as mean ± standard deviation and inter-quartile range. We used the Wilcoxon Rank-Sum test for numerical data. Categorical data were compared by chi-square test or by Fisher's exact test if appropriate. All tests are two-tailed and were performed by SPSS 15.0 software (SPCC Inc., Chicago, Illinois).

### Confidentiality and ethical aspects

This study protocol was approved by the central ethics committee of the Cliniques Universitaires UCL de Mont-Godinne (Intern identification number:78/2009) and it respects principles of the Helsinki Statement and the Belgian law for Good Clinical Practices from May 7th, 2004 relative to experiment on humans.

## Results

Overall, 114 patients were included in the study. During the 20-month period of the study, the total number of admission was 26,727 and the number of hospitalisation-days was 198,476. The number of routine microbiological samples analysed during this period of time was 53,545 (urinary analyses: 14,342, blood cultures: 17,339, respiratory tract samples: 8,782 and wounds or swabs: 13,082). The prevalence of ESBL-producing *Enterobacteriacae *isolates recovered from clinical samples (excluding duplicate isolates from the same patient) was 4.5% [114/2525]. The incidence rate of colonization/infection caused by ESBL-positive *Enterobacteriaceae *isolates was 4.3 cases/1000 admitted patients and 0.57 cases/1000 hospitalization days.

Data of the 114 patients were categorized according to the probable healthcare-associated or community origin of acquisition of ESBL-producing *Enterobacteriaceae*. Because of the study design and of the hospital-case mix of the population (85-90% of clinical specimens submitted to the laboratory originated from hospitalized patients), the healthcare-associated ESBL+ group was the most important (n = 104 [91%]) while the community acquired ESBL+ group was smaller (n = 10 [9%]). Compared with the group of patients with healthcare-associated ESBLs, patients with community-acquired ESBLs originated mostly from the ambulatory clinic (n = 7), were significantly younger (52 ± 20 years; P < 0.001) and were more frequently asymptomatic carriers (*Escherichia coli *(n = 8) harbouring CTX-M1 group ESBL) (*P*: 0.005).

The most salient clinical characteristics of the 104 patients with healthcare-associated colonization or infection are presented in Table 1. Their mean age was 70 ± 13 years (inter-quartile range: 62-79) with a sex ratio men/women of 1.2. Two-thirds of them were over 65 years of age. Twenty-four percent of the subjects in this subgroup were living in a nursing home (NH). At the time of microbiological sampling, 89 patients (86%) were hospitalized and 15 subjects (14%) were ambulatory care patients.

**Table 1:** Clinical data of patients with a healthcare-associated colonization or infection by an Extended-Spectrum Beta-Lactamase (ESBL) producing microorganism

Characteristics of patients	Healthcare-associated group N = 104 (91%)
**Demographic characteristics:**	
Male sex	56 (54)
Age: median; years (inter-quartile range)	71 (62-79)
Patients over 65 years	67 (64)
Nursing home residents, n (% of patients over 65 years)	16 (24)

**Proportion of ambulatory or hospitalized patients:**	
Outpatients	15 (14)
Inpatients	89 (86)

**Co-morbidities:**	
CIRS*: mean global score ± SD (inter-quartile range)	10.5 ± 3.9 (8-13)
CIRS: mean nr of different categories ± SD (inter-quartile range)	5.2 ± 2.0 (4-7)
Mean Norton Index ± SD;/20 points (inter-quartile range)	14.8 ± 3.6 (12-18)
Diabetes Mellitus	24 (23)
Chronic renal insufficiency	24 (23)
Heart failure	13 (12.5)
Severe chronic respiratory disease	17 (16)
Cirrhoses	3 (3)
Haematological malignancies	8 (8)
Pressure ulcers	16 (15)
Urinary catheters	38 (37)
Wounds	34 (33)
PEG** or tracheotomy	7 (7)

**Medications:**	
Proton pump inhibitors	34 (33)
Histamine_2 _receptor antagonist	25 (24)
Number of patients previously exposed to antibiotics:	60 (58)
Mean antibiotic use ± SD (inter-quartile range)	2 ± 1.5 (1-3)

**Clinical infection due to an ESBL microorganism:**	68 (65)
Urinary tract infection	38 (55.9)
Respiratory tract infection	18 (26.5)
Bloodstream infection	6 (8.8)
Others	6 (8.8)

**Simple ESBL carriers (colonization):**	36 (35)

**Previous known MRSA carriage:**	9 (9)

**Contact with health care system:**	
Previous hospitalization within 1 year	68 (65)
Multiple contacts within 1 year	71 (68)
Admission to ICU^§ ^during current hospitalization	30 (29)

**Median overall LOS^§§^, days (inter-quartile range):**	23 (13-39)
**Proportion with a long LOS (≥ 8 days)**	77 (87)

**Number of death:**	12 (12)

Data are provided in absolute number and percentage between brackets. * CIRS: Cumulative Illness Rating Scale for Geriatrics; **PEG: Percutaneous Endoscopic Gastrostomy; § ICU: Intensive Care Unit; §§ LOS: Length of hospital stay;

Hospitalized patients colonized or infected with an ESBL producer were admitted in all types of wards including surgery (n = 43), internal medicine (n = 17), intensive care unit (n = 11), oncology/haematology (n = 10), and geriatric wards (n = 8). A high number of co-morbid conditions were present in these patients (diabetes mellitus (23%), chronic renal insufficiency (23%), congestive heart failure (13%), chronic respiratory disease (16%), and haematological malignancies (8%)). The mean CIRS-G global score was 10.5 ± 3.9 (mean number of different categories: 5.2 ± 2.0) and the mean Norton Index was 14.8 ± 3.6. Intravascular, bladder catheter or gastrostomy tubes were present in half of the patients. Other risk factors commonly present for the acquisition of a MDR-bacteria were also found; two-thirds of the hospitalized patients had been previously exposed to antibiotics (with a mean of two different antibiotic regimens) prior to the isolation of an ESBL-producing organism, over 60% of them had been hospitalized or had had multiple medical contacts with the hospital within the last 12 months, and 29% of the patients had been hospitalized in the ICU during hospitalization before detection of an ESBL.

The list of antimicrobial agents prescribed in the time elapsed between hospital admission and the first known isolation of an ESBL-producing microorganism is reported in Table 2. Fifty-three percent (n = 52) of the patients had received cephalosporins, 32% (n = 30) had received a broad-spectrum penicillin (amoxicillin-clavulanate, piperacillin-tazobactam) and 13% (n = 12) had had a previous course of therapy with a fluoroquinolone agent.

**Table 2:** Antibiotic treatment courses administered prior to the isolation of an Extended-Spectrum Beta-Lactamase (ESBL) microorganism among hospitalized patients

Antibiotic treatment	ATC class	N (%)
Penicillins:	J01CE01	3 (3)
Aminopenicillins:	J01CA04/J01CA01	4 (4)
Carboxypenicillins:		
Temocillin:	J01CA17	3 (3)
Flucloxacillins:	J01CF05	5 (5)
Amoxicillin-clavulanate	J01CR02	14 (15)
Piperacillin-tazobactam:	J01CR05	16 (17)
Cephalosporins:		
Cefazolin:	J01DB04	22 (24)
Cefuroxime:	J01DC02	11 (13)
Ceftriaxone:	J01DD04	5 (5)
Ceftazidime:	J01DD02	5 (5)
Cefepime:	J01DE01	5 (5)
Meropenem:	J01DH02	3 (3)
Fluoroquinolones:	J01MA14/J01MA02	12 (13)
Aminoglycosides:	J01GB06/J01GB03	8 (9)

Sixty-eight (65%) of the hospitalized patients were considered as infected while 36 patients were asymptomatic carriers of an ESBL-producing *Enterobacteriaceae *isolate (35%). The four major documented infections were: urinary tract infection (UTI) (n = 38, [56%]), lower respiratory tract infection (RTI) (n = 18, [27%]), septicaemia (n = 6, [9%]) and intra-abdominal infections (n = 3, [4%]).

The median hospital length of stay (LOS) was 23 days (inter-quartile range: 13-39 days) and the overall mortality rate during hospitalization was 13% (Confidence Interval 95% [95% CI]: 7-19). During the study period, the average LOS in the institution was of 7.4 days while the number of death was 602 (2.3 cases/100 admitted patients).

A fatal outcome was documented only in patients with ESBL-producing bacteria of healthcare-associated onset (12 deceased patients out of 104 vs. 0 out of 10 in those with community acquired ESBL; *P *= 0.01). Compared to patients who were still alive at the end of their hospital stay, deceased patients had a significantly higher mean LOS (42 ± 30 days; *P: *0.05). Six out of the 12 patients who died were hospitalized in the ICU at the time of death. Of the 12 patients with a fatal outcome, 10 patients were infected (5 lower respiratory tract infection, 3 bloodstream infection and 2 urinary tract infection) while 2 only were ESBL carriers. However, mortality could be directly attributed to the infection in only 5 of the 10 cases.

The median time elapsed between hospital admission and the subsequent isolation of an ESBL-producing *Enterobacteriaceae *isolate was 7 days (inter-quartile range: 1-18 days). Microbiological data for healthcare-associated detection of ESBL-producing *Enterobacteriaceae *are shown in Table 3. *Escherichia coli *accounted as the most frequent species (n = 69, [66%]), followed by *Enterobacter *spp. (n = 21 [22%]: *E. aerogenes *(n = 14) and *E. cloacae *(n = 7)) and *Klebsiella *spp. (n = 12 [12%]: *K. pneumoniae *(n = 11) and *K. oxytoca *(n = 1)). ESBL enzymes of the CTX-M-1 group were the most frequently found (n = 51, [49%]) followed by TEM-types (n = 28, [27%]), SHV-types (n = 28, [27%]), CTX-M-9 (n = 8, [8%]) and CTX-M-2 groups (n = 6, [6%]). Enzymes of the CTX-M-1 group were essentially detected among *E. coli *while the other types of ESBLs were more frequent in other bacterial species. As anticipated, *E. coli *was more frequently found in association with urinary tract infections (32/38, [84%] in UTIs versus 14/30, [47%] in other types of infections; *P *< 0.001).

**Table 3:** Microbiological data for patients with a healthcare-associated colonization or infection by an Extended-Spectrum Beta-Lactamase (ESBL)-producing microorganism

Characteristics of patients	Healthcare-associated group N = 104 (91%)
**Proportion of clinical samples:**	
Urinary tract samples	59 (57)
Respiratory tract samples	22 (21)
Wounds swabs	11 (11)
Blood cultures	10 (10)
Others (stool, biopsy)	2 (2)

**Isolated microorganisms and types of ESBLs:**	
***Escherichia coli***	69 (66)
-CTX-M-1 group	45 (65)
-CTX-M-2 group	6 (9)
-CTX-M-9 group	5 (7)
-TEM	10 (14)
-SHV	3 (4)
***Enterobacter *spp**.	21 (22)
-CTX-M-9 group	2 (10)
-CTX-M-1 group	1 (5)
-TEM+SHV	14 (67)
-TEM	2 (10)
+SHV	2 (10)
***Klebsiella *spp**.	12 (12)
-CTX-M-1 group	2 (17)
-CTX-M-1+SHV	1 (8)
-CTX-M-9 group	1 (8)
-TEM	2 (17)
+SHV	6 (50)
***Others***	2 (2)
**Time elapsed from admission to positive culture:**	
< 72 hours	30 (34)
3-7 days	12 (13)
8-14 days	19 (21)
> 14 days	28 (31)

## Discussion

This study reports the clinical patterns in a cohort of subjects carrying ESBL-producing *Enterobacteriaceae *isolates over a 20-month period. We found that a two-thirds of the patients carrying ESBLs were elderly adults aged over 65 years and who presented a large variety of co-morbidities (on the average, 5 ± 2 different organs affected per patient), as illustrated by the high mean score of the CIRS-G index. Interestingly, the majority of these patients (whether being asymptomatic carriers or whether presenting an ESBL infection) had had several hospitalizations or multiple contacts with the hospital within the previous 12 months. The proportion of patients who had received one or more courses of antimicrobial therapy before the isolation of an ESBL-positive strain was high (58%, n = 60). It is most probable that the frequent exposure to previous antimicrobial treatment played a major role in the selection of ESBL-producing organisms as previously demonstrated in several case-control studies [6,20-22]. Further, antibiotic exposure is also known as a risk factor for the development of community-acquired bacteraemia caused by ESBL-producing *E. coli *[7], but due to the small number of patients with true community-acquired ESBL infection in our cohort we were not able to confirm this in the present study. In their comprehensive review of the literature, Pitout *et al. *highlighted the previous usage of antimicrobial agents (in particular cephalosporins), the severity of illnesses, a longer duration of hospital stay especially in the intensive care unit and the presence of urinary or arterial catheterisation as independent risk factors for the development of health-care associated ESBL infections [8].

One recent study in France, also highlighted the importance of inter-hospital transfer, especially after hospitalization in a foreign country, the previous carriage of an ESBL-producing *Enterobacteriaceae *or the presence of a chronic illness as risk factors for the acquisition of ESBLs [23]. Interestingly, these investigators also found that 4% of admitted patients without any potential risk factors were ESBL+ at entry.

The retrospective design of our study did not allow to establish the risk of co-colonization by ESBL-producing gram-negative bacilli and by other drug resistant bacterial pathogens such as methicillin-resistant *Staphylococcus aureus *(MRSA) or vancomycin-resistant *Enterococci *(VRE). However it was interesting to note that 9% (n = 9) of the patients colonized by an ESBL-producing organism were also known as previous MRSA carriers. The risk of co-colonisation probably reflects the occurrence of common risk factors for colonization or infection by different types of nosocomial pathogens [24].

The median LOS of 23 days and the 13% mortality rate observed here appear as much higher figures than the expected average rates observed at our institution (7.4 days and 2.3 death/100 admitted patients respectively). Lautenbach et *al. *also reported a longer LOS (OR: 1.74) and they documented higher hospital costs (OR: 2.9) associated with infections caused by an ESBL-producing *E. coli *or *K. pneumoniae *organism in comparison to a matched-control group of patients infected with organisms belonging to these same species but that were not producing an ESBL [25] . Furthermore, a positive association has also been reported between the LOS and the risk of colonization by an ESBL-producing microorganism. For instance, Friedmann et *al. *found in a non-outbreak setting that one-third of patients in general medicine wards (mean age of 76 ± 13 years), were colonized by ESBL-producing *Enterobacteriaceae *after a course of hospitalization of 2 weeks [12].

Several case-control studies have suggested that infections caused by ESBL-producing *Enterobacteriaceae *species were associated with a higher mortality rate as compared to infections caused by the same organisms but lacking an ESBL [26-28]. A recent meta-analysis concluded that bacteraemia caused by ESBL-producing *Enterobacteriaceae *was associated with an increased mortality rate (pooled Relative Risk [RR]: 1.85, 95%CI: 1.39-2.47, *P *< 0.001) and that delayed effective therapy did further increase the mortality (pooled RR: 5.56, 95% CI: 2.94-10.51, *P *< 0.001) [29]. Nevertheless, the real attributable impact of ESBL infection on mortality remains controversial and a critical review of the literature does not provide clear answers to this matter [28]. The lack of well-powered studies, the importance and clinical severity of the underlying illnesses, the appropriate or inappropriate choice of empirical therapy, as well as variable performance of microbiology laboratories to detect and report ESBL microorganisms, might all be limiting factors or confounding variables for a correct interpretation of the data. Well designed prospective studies are thus clearly warranted to address this important issue [28].

As anticipated, we found that *E. coli *by far accounted as the most frequent ESBL-producing enterobacteria species. A recent study carried out in 2004 and involving 16 laboratories in London and in the South-Eastern part of England showed that among 1127 cephalosporin-resistant gram-negative isolates from both hospital and community settings, *E. coli *also accounted as the largest group (51%) and, in particular, CTX-M producing strains [30]. The high prevalence of CTX-M-1 group ESBLs (mainly comprising the CTX-M-15 type) is an emerging and well described problem worldwide [31-37]. While most published reports until the 1990's found a predominance of SHV- or TEM-type ESBLs associated with nosocomial outbreaks, the epidemiology dramatically changed over the last ten years. The extensive dissemination of *E. coli *in the community [37] and its subsequent spread in hospitals illustrates the importance of movement of patients between the community and the healthcare system, in particular between nursing homes and acute hospitals. Data from our study further highlight this emerging pattern. In France, a progressive increase in the occurrence of CTX-M enzymes linked to *E. coli *has also been reported [31,37]. In Belgium, a three-fold increase of the prevalence of multi-resistant CTX-M producing *E. coli *was detected between 2000 and 2004 [38] and over 50% of the ESBL-producing *Enterobacteriaceae *isolates identified in human clinical isolates in Belgium in 2008 belong to the CTX-M [39]. CTX-M-15 (75% of all CTX-M ESBLs) and CTX-M-2 to a lesser extent currently account as the most predominant ESBLs of the CTX-M type in Belgium and this likely reflects both clonal dissemination as well as plasmid epidemics across different bacterial species (*Salmonella enterica *and *E. coli*) in animals and in humans [40].

There have been only few studies which did recently address the epidemiology of ESBL-producing *Enterobacteriaceae *in Belgium, and the present report gives some insights on the clinical patterns of healthcare associated colonization or infection caused by ESBL-producing *Enterobacteriaceae*.

We do however recognize the limitations of this retrospective study which was only based on the collection of routine microbiological samples with no screening being carried out to detect asymptomatic carriage upon hospital admission. Comparing and translating data obtained from studies carried out in epidemic or endemic settings with a systematic screening approach may therefore be difficult.

Further, we acknowledge the fact that the selection of patients from clinical samples submitted to a hospital-based laboratory may have introduced some bias. Indeed, almost 90% of the clinical specimens submitted for microbiological analysis to our bacteriology laboratory originate from hospitalized patients and/or from subjects with frequent contacts with the healthcare system. This may be explained by the fact that our hospital is geographically located in a rural area and that over 90% of the microbiological specimens received in our laboratory originates either from hospitalized patients or from patients regularly visiting the polyclinics; thus the group of patients with true community acquired infections clearly constitutes a minor proportion in our setting.

The lack of a control group constitutes a limiting factor excluding possible comparisons of odds ratios for clinical risk factors.

Despite all the limitations we think these data may be interesting for the following reasons: Apart from of one recent microbiological report of ESBLs isolated from humans in Belgium [40], our study constitutes one of the first attempt of a global clinical description of a large cohort of patients colonized or infected by ESBL-producing gram-negative bacilli at one university hospital. Since the majority of the patients were aged over 65 years, the patient's characteristics were also defined with geriatric tools such as the Cumulative Illness Rating Scale for Geriatrics and the Norton Index. The reliance on these objective scales and indexes may be useful to develop algorithms and screening strategies aiming to select at risk groups in this population upon hospital admission. Urinary tract and respiratory tract infections were the two major diagnostic categories and these indeed constitute the most frequent types infections among elderly patients admitted in general internal medicine or geriatric wards. While most clinicians do currently recognize the importance of MRSA screening upon hospital admission, few teams would consider assessing the potential risk factors for ESBL carriage upon hospital admission. Nevertheless, asymptomatic intestinal carriage of ESBLs could represent as for MRSA an important risk factor for the development of subsequent infections. One study carried out in a large cohort of elderly patients screened upon hospital admission in Israel showed an increase rate of subsequent bacteraemia for ESBL faecal carriers (15% versus 0.5%; odds ratio: 38.9; *P *< 0.001) [5]. A more comprehensive approach may also lead to improvement in the choice of empiric antibiotic treatment, infection control procedures and patient outcomes.

We strongly believe that multidisciplinary work involving geriatricians, infectious diseases specialists, infection control units and the microbiological laboratory could improve clinical practice and help reducing antibiotic pressure [41].

## Conclusion

Prospective studies with systematic screening of asymptomatic ESBL carriage in subjects residing in nursing homes and in elderly patients upon admission to hospitals in geriatric wards are necessary to improve our knowledge on the dynamic of acquisition and transmission of ESBLs between different human sectors.

## Competing interests

None of the authors had any financial interest or support for this paper. Authors disclose any financial and non-financial competing interests for the preparation and publication of this manuscript. No specific funding was provided for the current study.

## Authors' contributions

DS: did review all microbiological/demographic and clinical data, analyse of the data, did write the article. PB: was involved in microbiological analyses and revision of the manuscript.

AG was involved in study design, extraction of data and review of the manuscript. BD and MSH were involved in full revision of the manuscript. CS: was involved in supervision of the research process, elaboration of the study design and full correction of the manuscript. JJ: involved in statistical analyses and revision of the manuscript. YG: involved in study conception and design, critical discussion, writing and review of the manuscript. All authors have read and approved the final manuscript.

## Pre-publication history

The pre-publication history for this paper can be accessed here:

http://www.biomedcentral.com/1471-2334/11/12/prepub
